# Smoking and Visual Impairment Among Older Adults With Age-Related Eye Diseases

**Published:** 2011-06-15

**Authors:** Xinzhi Zhang, Jennifer Kahende, Amy Z. Fan, Yan Li, Lawrence Barker, Theodore J. Thompson, Jinan B. Saaddine, Ali H. Mokdad

**Affiliations:** National Center for Chronic Disease Prevention and Health Promotion, Centers for Disease Control and Prevention; Office on Smoking and Health, National Center for Chronic Disease Prevention and Health Promotion, Centers for Disease Control and Prevention, Atlanta, Georgia; Division of Adult and Community Health, National Center for Chronic Disease Prevention and Health Promotion, Centers for Disease Control and Prevention, Atlanta, Georgia; Division of Adult and Community Health, National Center for Chronic Disease Prevention and Health Promotion, Centers for Disease Control and Prevention, Atlanta, Georgia; Division of Diabetes Translation, National Center for Chronic Disease Prevention and Health Promotion, Centers for Disease Control and Prevention, Atlanta, Georgia; Division of Diabetes Translation, National Center for Chronic Disease Prevention and Health Promotion, Centers for Disease Control and Prevention, Atlanta, Georgia; Division of Diabetes Translation, National Center for Chronic Disease Prevention and Health Promotion, Centers for Disease Control and Prevention, Atlanta, Georgia; Institute for Health Metrics and Evaluation, University of Washington, Seattle, Washington

## Abstract

**Introduction:**

Tobacco use is the leading preventable cause of death in the United States. Visual impairment, a common cause of disability in the United States, is associated with shorter life expectancy and lower quality of life. The relationship between smoking and visual impairment is not clearly understood. We assessed the association between smoking and visual impairment among older adults with age-related eye diseases.

**Methods:**

We analyzed Behavioral Risk Factor Surveillance System data from 2005 through 2008 on older adults with age-related eye diseases (cataract, glaucoma, age-related macular degeneration, and diabetic retinopathy; age ≥50 y, N = 36,522). Visual impairment was defined by self-reported difficulty in recognizing a friend across the street or difficulty in reading print or numbers. Current smokers were respondents who reported having smoked at least 100 cigarettes ever and still smoked at the time of interview. Former smokers were respondents who reported having ever smoked at least 100 cigarettes but currently did not smoke. We used multivariate logistic regressions to examine the association and to adjust for potential confounders.

**Results:**

Among respondents with age-related eye diseases, the estimated prevalence of visual impairment was higher among current smokers (48%) than among former smokers (41%, *P* < .05) and respondents who had never smoked (42%, *P* < .05). After adjustment for age, sex, race/ethnicity, education, and general health status, current smokers with age-related eye diseases were more likely to have visual impairment than respondents with age-related eye diseases who had never smoked (odds ratio, 1.16, *P* < .05). Furthermore, respondents with cataract who were current smokers were more likely to have visual impairment than respondents with cataract who had never smoked (predictive margin, 44% vs 40%, *P =* .03), and the same was true for respondents with age-related macular degeneration (65% of current smokers vs 57% of never smokers, *P =* .02). This association did not hold true among respondents with glaucoma or diabetic retinopathy.

**Conclusion:**

Smoking is linked to self-reported visual impairment among older adults with age-related eye diseases, particularly cataract and age-related macular degeneration. Longitudinal evaluation is needed to assess smoking cessation's effect on vision preservation.

## Introduction

Tobacco use causes approximately 443,000 deaths annually and is the leading preventable cause of death in the United States (1). Smoking harms nearly every organ of the body, causes many diseases, and worsens the general health of smokers. Moreover, secondhand smoke causes diseases and premature death in nonsmoking children and adults (2). Smoking annually results in an estimated $96 billion in direct costs and $97 billion in productivity losses (1). Lifetime additional direct medical expenditures among smokers were $16,454 for men and $19,275 for women in 2004 (3).

Blindness and visual impairment are among the 10 most common causes of disability in the United States (4) and are associated with shorter life expectancy and lower quality of life (5,6). In 2000, blindness or low vision, mainly caused by age-related eye diseases (ARED, including cataract, glaucoma, age-related macular degeneration [AMD], and diabetic retinopathy [DR]), affected more than 3.3 million Americans aged 40 years or older; this number is predicted to increase more than 50% by 2020 (7). The annual economic effect of major vision problems among the adult population aged 40 years or older was more than $51 billion in 2004 (8,9).

Many studies have explored the associations between smoking and ARED. The 2004 Surgeon General's report on smoking concluded that a causal relationship between smoking and nuclear cataract exists and found evidence that was suggestive of a relationship between smoking and AMD (10). The report suggested an absence of a causal relationship between smoking and DR and found that evidence of a relationship between smoking and glaucoma was not conclusive (10). ARED is strongly associated with visual impairment (7). However, the relationship between smoking and visual impairment has not been extensively assessed and is not clearly understood. We assessed the cross-sectional association between smoking and visual impairment among older adults (age ≥50 y) with ARED by using data from the Behavioral Risk Factor Surveillance System (BRFSS).

## Methods

### Data source

BRFSS is a state-based, random-digit–dialed telephone survey of the noninstitutionalized, US civilian population aged 18 years or older. With approximately 350,000 adults participating each year, BRFSS can produce local, state, and national estimates on health-related information, including chronic illness, health behaviors, and access to health care. Survey methods, questionnaires, data, and relevant reports can be found at www.cdc.gov/brfss.

**Table:** 

**Box. States That Included the Visual Impairment and Access to Eye Care Module, Behavioral Risk Factor Surveillance System, 2005-2008**	
2005	Iowa, Louisiana, Ohio, Tennessee, and Texas
2006	Arizona, Florida, Georgia, New York, Ohio, Tennessee, and Texas
2007	Alabama, Georgia, Iowa, New York, and West Virginia
2008	Alabama, Connecticut, Indiana, Missouri, New Mexico, North Carolina, Tennessee, and Wyoming

The BRFSS questionnaire consists of 3 sections each year: core sections, optional modules, and state-added questions. The BRFSS vision module (Visual Impairment and Access to Eye Care) was first administered in 2005 among participants aged 50 years or older. We analyzed available data from the pooled sample of 36,522 participants aged 50 years or older with ARED by using the vision module from 2005 through 2008 (n = 5,108 in 2005; n = 10,061 in 2006; n = 7,186 in 2007; and n = 14,167 in 2008). During 2005 through 2008, the median BRFSS response rate for states that included the vision module (Box) was 52.7%, and ranged from 34.0% to 61.5%.

### Assessment of vision impairment and smoking status

Visual impairment was assessed in the vision module with the questions, "How much difficulty, if any, do you have in recognizing a friend across the street," and "How much difficulty, if any, do you have reading print in newspaper, magazine, recipe, menu, or numbers on the telephone?" We classified respondents as having visual impairment if they answered "a little difficulty," "moderate difficulty," "extreme difficulty," or "unable to do because of eyesight" to either question. If respondents usually wear glasses or contact lenses, they were asked to rate their ability to do these activities while wearing glasses or contact lenses.

Current smokers were respondents who reported smoking at least 100 cigarettes during their lifetimes and reported continuing to smoke at the time of the interview. Former smokers were those who reported ever smoking at least 100 cigarettes but not smoking at the time of the interview. Never smokers were respondents who reported smoking fewer than 100 cigarettes in their lifetimes and did not smoke at the time of the interview.

### Other variables

We identified 3 eye diseases (cataract, glaucoma, and AMD) among respondents who answered yes to the questions in the vision module, "Have you ever been told by an eye doctor or other health care professional that you had (cataract, glaucoma, or AMD)?" We identified DR (from the BRFSS diabetes module) if respondents with diabetes answered yes to the question, "Has a doctor ever told you that diabetes has affected your eyes or that you had retinopathy?"

We also included age (50-64 y, ≥65 y), sex (male, female), race/ethnicity (non-Hispanic white, non-Hispanic black, Hispanic, other), educational attainment (<high school graduate, high school graduate, some post–high school education), and self-assessed general health status (poor or fair, good, very good or excellent).

### Statistical analysis

We used SAS-callable SUDAAN version 10.0 (Research Triangle Institute, Research Triangle Park, North Carolina) to account for the complex sampling design of BRFSS. Respondents from the states that implemented 2 years of the vision module were given half the weight each year. All estimates were weighted to represent the sampled population. We assessed the association between smoking and visual impairment after adjusting for age, sex, race/ethnicity, educational attainment, and general health status. We performed logistic regression to examine the associations between smoking status and visual impairment to calculate odds ratios (ORs) and corresponding 95% confidence intervals (CIs). Results were considered significant at *P* < .05.

We further examined independent associations between smoking and visual impairment among people with each ARED (cataract, glaucoma, AMD, DR). After controlling for other independent variables, we used multivariate logistic regressions to estimate the probability of having visual impairment among people with each ARED. We used Taylor linearization to estimate predictive margins (PMs) and their 95% CIs. Missing values were not included in the models but were accounted for in our variance estimation.

## Results

Demographic and health status characteristics of current smokers, former smokers, and never smokers with ARED varied significantly (Table 1). Current smokers were younger than former and never smokers, and the proportion of non-Hispanic whites was higher among former smokers than current smokers and never smokers. We found no significant difference in the sex composition of former smokers, although more current smokers and never smokers were female than male. Education level was higher among former smokers and respondents who had never smoked than among current smokers. More former smokers and never smokers considered their health status as "excellent or very good" compared with current smokers.

Prevalence of visual impairment among respondents with ARED was significantly higher among current smokers (48%) than among former smokers (41%, *P* < .05) and never smokers (42%, *P* < .05) (Table 2). Compared with never smokers with ARED, current smokers with ARED had higher unadjusted odds of visual impairment (OR, 1.31). After adjustment for age, sex, race/ethnicity, education levels, and general health status, current smokers with ARED were more likely than never smokers with ARED to have visual impairment (OR, 1.16, *P* < .05), and odds of being visually impaired were similar between former smokers and never smokers (OR, 1.04).

Among respondents with cataract, the predictive margin (PM) of having visual impairment was higher among current smokers than never smokers (44% vs 40%, *P* = .03) (Table 3). Among respondents with AMD, current smokers were also more likely to be visually impaired compared with never smokers (65% vs 57%, *P* = .02). The association was not significant among respondents with glaucoma and DR.

## Discussion

We found that smoking is associated with self-reported visual impairment among older adults with ARED, especially those with cataract and AMD. Smoking may be linked to ocular diseases and conditions including cataract, AMD, Graves' ophthalmopathy, ocular irritation, and ocular ischemia (11). However, few studies have addressed the association between smoking and visual impairment. A New Zealand study suggested that the blindness of 26.8% of registered blind people aged 55 years or older with AMD and 9.5% of those with cataract was attributable to smoking (12). Results from the Canadian Study of Health and Aging suggested that the odds of self-reported visual impairment were 2.8 times as high among smokers than nonsmokers (13). Although we did not find odds this large, our findings suggested that smoking was independently associated with visual impairment among older adults with ARED (current smokers vs never smokers).

Smoking is strongly associated with the development of cataract, particularly nuclear cataract. Weintraub et al found that, compared with current smokers, former and nonsmokers were less likely to have cataract extraction (14). Recent findings from the Visual Impairment Project and the Blue Mountains Eye Study confirmed a higher risk and dose response of smoking and having nuclear cataract (15,16). We also found an independent significant association between smoking (current smokers vs never smokers) and visual impairment among those with cataract. However, we did not find a significant difference between former smokers and never smokers.

Our findings suggested a strong association between smoking and visual impairment among people with AMD that is consistent with findings in the British population. After controlling for potentially confounding factors, current smokers were twice as likely to have AMD that caused visual impairment compared with nonsmokers in a British study; for former smokers, the association was not significant (17). We found that visual impairment among BRFSS respondents with AMD was higher among current smokers than never smokers. Smoking seemed to be associated with more severe AMD cases. Similarly, the Beaver Dam Eye study suggested that, after controlling for age, sex, and baseline AMD severity, current smokers were at higher risk of progression of AMD than nonsmokers (18). The Blue Mountains Eye Study found that current smokers had an increased risk of 5-year incidence of late age-related maculopathy lesions and developed maculopathy at a significantly earlier age (19). These findings suggest a possible effect of smoking on progression of AMD.

Although the 2004 Surgeon General's report found insufficient evidence to infer a causal relationship between smoking and AMD, more recent studies indicate a strong association between current smoking and AMD. Smoking was associated with an increased frequency of recurrence of exudative AMD after laser photocoagulation, the only proven treatment for AMD (20). According to a recent systematic review, the evidence meets the criteria for causality (21). Recent findings from large population-based studies such as the Beaver Dam Eye Study also indicated an increased risk of incident early AMD during a 15-year follow-up (18). Furthermore, the number of pack years of smoking was found to be strongly associated with AMD (22).

Our findings differ from those of the Beaver Dam Eye Study, which suggested that smoking plays a minor role in primary open-angle glaucoma (POAG) (23). A meta-analysis of 7 reports (4 cross-sectional studies and 3 case-control studies) before 2003 suggested a pooled odds of POAG of 1.37 for current smokers and 1.03 for former smokers (24). However, a recent systematic review of case-control and cohort studies by Edwards et al concluded that little evidence exists for a causal association between smoking and development of POAG (25).

Some findings suggest a role of smoking in poorer glycemic control (elevated hemoglobin A1c levels), escalating insulin resistance, and an increase in microvascular complications (eg, microalbuminuria) (26-28). Furthermore, current smokers with type 1 diabetes have higher odds of severe hypoglycemia than patients with type 1 diabetes who do not smoke (29). A prospective cohort study of CARDIA (coronary artery risk development in young adults) indicated that both active and passive smoke exposure play a role in developing glucose intolerance (including impaired fasting glucose and diabetes) (30). Although the literature in general suggested little or no association between cigarette smoking and the incidence or progression of DR (10), Mouton and Gill found that smoking influences the severity of DR (31). In this study, we found no significant relationship between smoking and visual impairment among BRFSS respondents with DR.

In summary, smoking is a major modifiable risk factor for AMD (11), and smoking is the most important modifiable risk factor for primary and secondary prevention of cataract (10,11). A lack of awareness may exist among health care providers and patients about the risks of developing eye diseases and vision loss from smoking. Of an estimated 61 million adults in the United States who are at high risk for serious vision loss due to aging, diabetes, or vision or eye problems, only half visit an eye care provider annually, making the situation even worse (32). Given the effect of smoking on a person's overall health, and especially on sight, comprehensive tobacco control interventions, as recommended by the Centers for Disease Control and Prevention (CDC), are needed (10,33). These interventions include health care provider counseling, telephone quit lines, insurance coverage for cessation therapies, legislation of clean indoor air, and increased tobacco taxes (33).

This research is subject to several limitations. First, the prevalences of visual impairment and eye diseases are self-reported and may differ from objective clinical measurements. Data on family history of eye diseases were unavailable. Social desirability bias may have caused some current or former smokers to identify themselves as former or never smokers. Accordingly, the actual effect of smoking may be larger than what we found. Second, the data were collected by telephone survey and may not be representative of people without landline telephones. Although low, the BRFSS response rate is in the normal range for telephone surveys. BRFSS data are valid and reliable when compared with other household surveys (34). Third, institutionalized populations (eg, nursing home residents) are not included in the BRFSS. Fourth, only a few states used the BRFSS vision module, so our findings may not be nationally representative. Fifth, data on frequency and quantity of tobacco use were not obtained and could not be analyzed. Finally, data were cross-sectional; therefore, we were unable to identify causal relationships.

In conclusion, self-reported smoking is linked to self-reported visual impairment among older adults with ARED. The associations between smoking and visual impairment were mostly observed among BRFSS respondents with cataract and AMD but not among those with glaucoma and DR. Further longitudinal evaluation is warranted to explore how smoking cessation or prevention might benefit vision preservation.

## Figures and Tables

**Table 1:** Characteristics of Study Population With Age-Related Eye Diseases, by Smoking Status, Behavioral Risk Factor Surveillance System, 2005-2008^a^

**Characteristic**	Current Smokers (n = 4,517), %^b^ (95% CI)	**Former Smokers (n = 14,165), % (95% CI)**	**Never Smokers (n = 17,651), % (95% CI)**
**Age, y**			
50-64	52.9 (50.3-55.6)	25.9 (24.5-27.4)	27.9 (26.6-29.2)
≥65	47.1 (44.4-49.7)	74.1 (72.6-75.5)	72.1 (70.8-73.4)
**Sex**			
Male	44.1 (41.4-46.9)	51.1 (49.6-52.5)	27.0 (25.8-28.3)
Female	55.9 (53.1-58.6)	49.0 (47.5-50.4)	73.0 (71.7-74.2)
**Race/ethnicity**			
Non-Hispanic white	77.0 (74.5-79.4)	83.1 (81.9-84.3)	77.0 (75.7-78.3)
Non-Hispanic black	9.8 (8.4-11.4)	7.7 (7.0-8.5)	9.7 (8.9-10.6)
Hispanic	7.5 (5.9-9.4)	5.5 (4.8-6.4)	9.1 (8.2-10.1)
Other	5.7 (4.4-7.4)	3.7 (3.1-4.3)	4.1 (3.5-4.8)
**Education**			
Less than high school graduate	21.3 (19.3-23.5)	15.9 (14.8-17.0)	16.6 (15.5-17.6)
High school graduate	36.3 (33.8-38.9)	32.0 (30.7-33.4)	34.1 (32.9-35.4)
Some post–high school education	42.4 (39.7-45.0)	52.1 (50.6-53.6)	49.3 (48.0-50.7)
**Self-assessed general health status**			
Excellent or very good	24.6 (22.4-27.0)	32.6 (31.2-33.9)	35.1 (33.8-36.4)
Good	30.8 (28.4-33.3)	32.3 (30.9-33.7)	33.4 (32.1-34.7)
Fair or poor	44.6 (41.9-47.3)	35.2 (33.7-36.6)	31.5 (30.2-32.8)

Abbreviation: CI, confidence interval.

a Current smokers were respondents who reported smoking at least 100 cigarettes during their lifetimes and reported continuing to smoke at the time of the interview, former smokers were those who reported ever smoking at least 100 cigarettes but not smoking at the time of the interview, and never smokers were respondents who reported smoking fewer than 100 cigarettes in their lifetimes and did not smoke at the time of the interview. Values for n do not sum to 36,522 because some respondents did not answer the question regarding smoking status.

b Percentages were weighted to population characteristics.

**Table 2:** Association Between Smoking and Visual Impairment Among People With Age-Related Eye Diseases, Behavioral Risk Factor Surveillance System, 2005-2008

** Type of Smoker^a^**	Prevalence,^b^ % (95% CI)	**Unadjusted OR (95% CI)**	Adjusted OR^c^ (95% CI)
**Current**	48.2 (45.6-50.9)	1.31 (1.16-1.48)	1.16 (1.02-1.32)
**Former**	41.2 (39.7-42.6)	0.98 (0.91-1.07)	1.04 (0.95-1.13)
**Never**	41.6 (40.2-42.9)	1 [Reference]	1 [Reference]

Abbreviations: CI, confidence interval; OR, odds ratio.

a Current smokers were respondents who reported smoking at least 100 cigarettes during their lifetimes and reported continuing to smoke at the time of the interview, former smokers were those who reported ever smoking at least 100 cigarettes but not smoking at the time of the interview, and never smokers were respondents who reported smoking fewer than 100 cigarettes in their lifetimes and did not smoke at the time of the interview.

b Estimated crude rate of visual impairment among participants who were current, former, or never smokers.

c Adjusted for age, sex, race/ethnicity, education levels, and general health status.

**Table 3:** Predictive Margin (Probability [%]) of Visual Impairment Among People With Age-Related Eye Diseases (Cataract, Glaucoma, AMD, or DR), by Smoking Status, Behavioral Risk Factor Surveillance System, 2005-2008

**Type of Smoker^a^**	**Cataract (n = 30,836), PM (95% CI)**	**Glaucoma** (n = 5,622), PM (95% CI)	**AMD** (n = 4,943), PM (95% CI)	**DR** (n = 2,883), PM (95% CI)
**Current**	44.0 (41.2-46.9)	45.1 (38.0-52.2)	65.4 (59.2-71.5)	56.9 (49.4-64.4)
**Former**	41.2 (39.6-42.8)	47.9 (44.4-51.5)	60.1 (56.4-63.9)	58.0 (53.0-63.0)
**Never**	40.4 (39.0-41.9)	46.6 (43.0-50.1)	56.7 (52.9-60.4)	51.9 (47.0-56.8)
***P* Value^b^**	.03	.72	.02	.27

Abbreviations: PM, predictive margin; CI, confidence interval; AMD, age-related macular degeneration; DR, diabetic retinopathy.

a Current smokers were respondents who reported smoking at least 100 cigarettes during their lifetimes and reported continuing to smoke at the time of the interview, former smokers were those who reported ever smoking at least 100 cigarettes but not smoking at the time of the interview, and never smokers were respondents who reported smoking fewer than 100 cigarettes in their lifetimes and did not smoke at the time of the interview.

b *P* values calculated by using *t* test, comparing current vs never smokers.
